# Semi-quantitative analysis of multiple chemical mixtures in solution at trace level by surface-enhanced Raman Scattering

**DOI:** 10.1038/s41598-017-06543-y

**Published:** 2017-07-21

**Authors:** Sumeng Zou, Mengjing Hou, Jianghao Li, Lingwei Ma, Zhengjun Zhang

**Affiliations:** 1 State Key Laboratory of New Ceramics and Fine Processing, School of Materials Science and Engineering, Tsinghua University, Beijing, 100084 P.R. China; 2 Key Laboratory of Advanced Materials (MOE), School of Materials Science and Engineering, Tsinghua University, Beijing, 100084 P.R. China

## Abstract

Surface-enhanced Raman scattering (SERS) technology combines with chemometric method of principal component analysis (PCA) was used to calculate the composition of chemical mixtures in solution. We reported here that there exists composition discrepancy between molecules in solution and molecules adsorbed on Ag@Al_2_O_3_ nanorods substrates due to difference in adsorption kinetics of each component. We proposed here a way to calculate the adsorption kinetics factor for each component using a standard sample as the reference, with which one could correct the predictions given by PCA. We demonstrate the validity of this approach in estimating the compositions of mixtures with two, three and four components of 1, 4-Benzenedithiol, 2-Naphthalenethiol, 4-Mercaptobenzoic acid, and 4-Mercaptopyridine molecules, with acceptable errors. Furthermore, a general formula applied to more complex mixtures was proposed to calculate compositions in solution.

## Introduction

Surface-enhanced Raman scattering (SERS) has long been considered as a powerful means for trace level detections of chemicals or molecules which should find potential applications in different fields, such as chemical sensing, food safety, environment monitoring, medical diagnosis, or drug delivery, etc^1–9^. The high sensitivity of SERS for trace level detections arises due to adsorption of close proximity of analytes to the surface of nanostructures of noble metals such as silver, gold or copper^10^, where their Raman cross sections can be largely enhanced due to the electromagnetic and chemical enhancements^11–19^. This, however, brings difficulty in quantifying analytes by SERS especially for the compositional analysis of chemical mixtures in solution, as the composition of chemical mixtures adsorbed on the surface of noble metals can be very different from that in the solution due to the difference in their adsorption kinetics.

Chemometrics methods, for example, the partial least squares regression (PLSR), the hierarchical cluster analysis (HCA), or principal components analysis (PCA), etc., have been employed to differentiate analytes components and correlate the amount by their SERS spectra^20–25^. For instance, Li *et al*. reported that SERS spectra of blood serum can be employed for differentiating healthy individuals and esophageal cancer patients by HCA and PCA^26^. Similarly, Feng *et al*. developed a technique based on SERS plasma analysis for cervical cancer detection in conjunction with PCA-LDA^27^. Luo *et al*. provided a method for quantitative analyzing phosment and thiabendazole in apples by PLSR models^28^. Moreover, Rivera-Betancourt *et al*. compared the validity of PCA, HCA, and PLS-DA in identifying mycobacteria utilizing mycolic acid Raman spectra^29^. Provided above achievements, quantification of chemical mixtures at trace level in solution remains still a challenge especially for the compositional analysis by SERS.

Recently, we performed and device a simple way to estimate the composition of chemicals adsorbed on Ag@SiO_2_ nanorods as the SERS substrate by PCA (which is normally used for discriminating molecules), and prove it experimentally through several trace-level chemical mixtures in solution, by neglecting the difference in their adsorption kinetics (i.e. treating the composition measured for chemicals adsorbed on the nanorods as the same in solution)^30^. This assumption is of course not correct for most chemical mixtures in solution, but it opens a simple and new route for quantitative analysis (specifically the compositional analysis) if we could find a way to properly consider the difference in the adsorption kinetics of chemicals in trace level solution.

We reported here that for trace-level multicomponent mixtures in solution, due to the difference in adsorption kinetics of each component, the composition of molecules adsorbed on Ag@Al_2_O_3_ nanorods as SERS substrate can be very different from that in solution. We proposed in this study a way to calculate the adsorption kinetics factor for each component using a standard sample as the reference, with which one could correct the predictions given by PCA, and demonstrated its success for binary, ternary, and quadruple chemical mixtures. This method might be developed into an effective quantitative SERS measurement approach of multicomponent mixtures at trace levels.

## Results and Discussions

**Figure 1:**
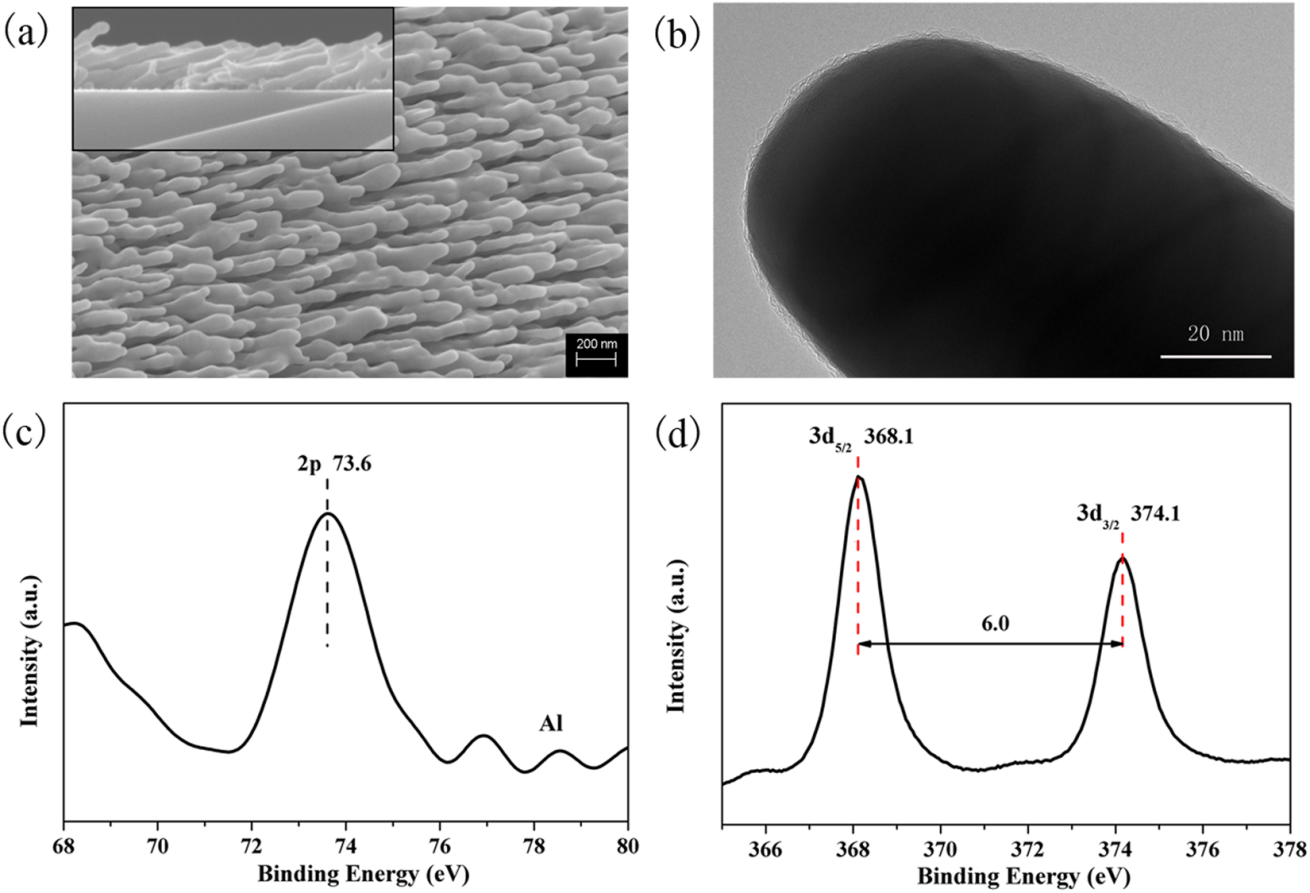
(**a**) Top-view SEM image of Ag@Al_2_O_3_ nanorods; inset is a corresponding side-view SEM image. (**b**) HRTEM image of the Ag@Al_2_O3 nanorods. (**c**) A typical Al 2p XPS spectrum of the Al_2_O_3_ layer. (**d**) Ag 3d_5/2_ and Ag 3d_3/2_ XPS spectra of Ag@Al_2_O_3_ nanorods.

Figure 1a shows the top-view SEM image of Ag@Al_2_O_3_ nanorods (inset represents the corresponding side-view SEM image). It is seen that the slanted Ag@Al_2_O_3_ NRs are well separated and highly oriented having length of 620 nm and diameter of 40 nm. Figure 1b represents the HRTEM image of the Ag@Al_2_O_3_ nanorods, showing that Ag nanorods are wrapped by Al_2_O_3_ which ensure the stable SERS sensitivity of substrates in air for few days. The typical Al 2p XPS spectrum (Fig. 1c) centered at 73.6 eV indicate the formation of Al-O bond in Al_2_O_3_^31^. Metallic properties of our samples were confirmed by the doublet Ag3d_5/2_ and Ag3d_3/2_ located at 368.1 eV and 374.1 eV(Fig. 1d), respectively^32, 33^ All these results proves that the Ag@Al_2_O_3_ nanorods possess the excellent SERS sensitivity and outstanding stability.

**Figure 2:**
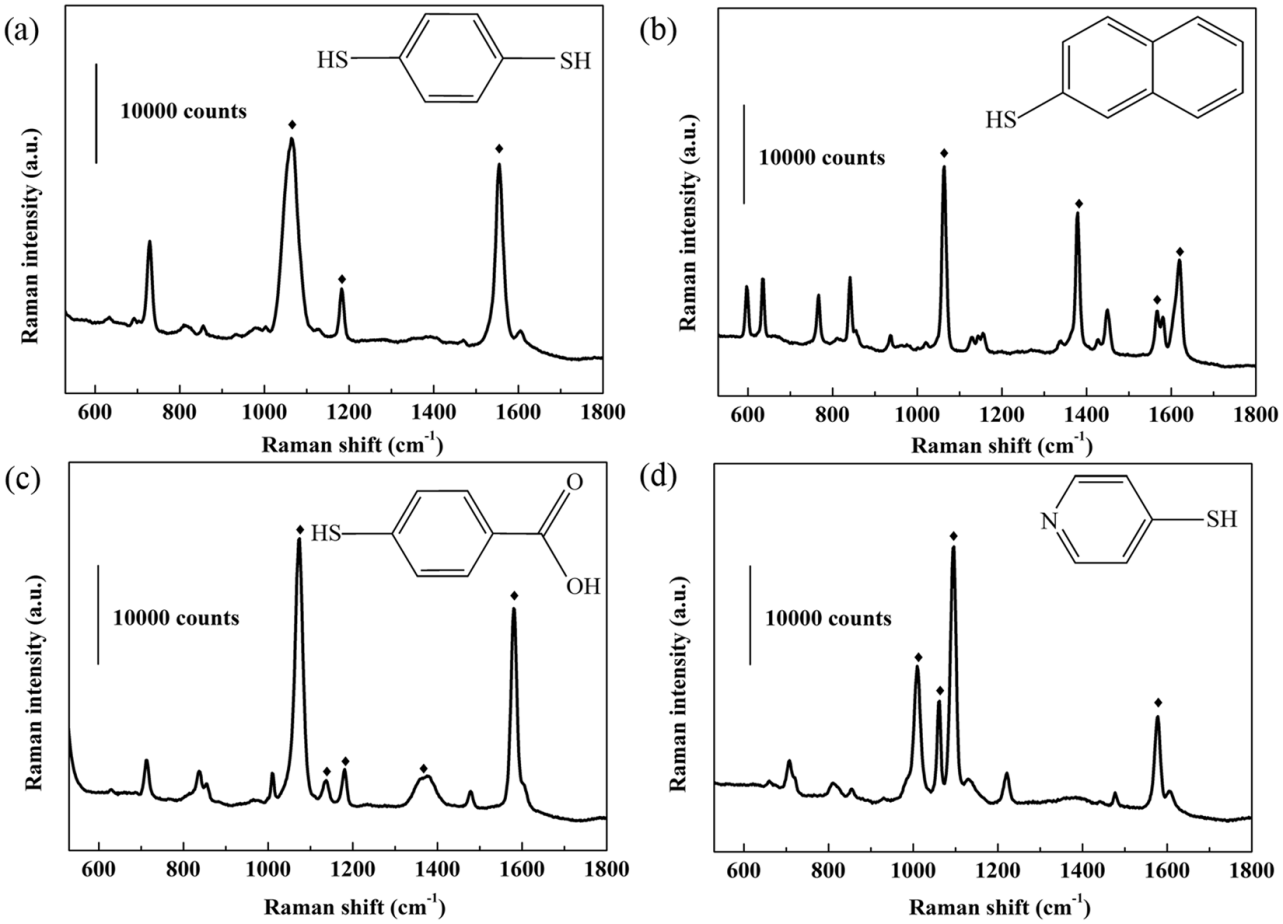
SERS spectrum of (**a**) 1, 4-BDT, (**b**) 2-NaT, (**c**) 4-MBA, and (**d**) 4-MPY.

Figure 2(a–d) shows the four probes molecule SERS spectra obtained on Ag@Al_2_O_3_ nanorod substrates at 1 × 10^−6^ M. These four molecules exhibited different spectra, and their spectral assignation was based on previous studies. The main peaks of 1,4-BDT at 1063, 1178 and 1563 cm^−1^ can be attributed to C–S stretching, C–H bending and C–C stretching respectively^34, 35^. For 2-NaT, the peaks at 1062, 1378, 1620, 1567 cm^−1^ are associated with C–H bending, ring stretching, C=C stretching, respectively^36–39^. The SERS peaks located at 1073, 1580 cm^−1^ are associated with the C-C stretching, 1135, 1178 cm^−1^ are related to C-H bending, and 1369 cm^−1^ arises from COO- stretching for 4-MBA^40–42^. The main Raman peaks of 4-MPY include ring breathing at 1007 cm^−1^ and 1094 cm^−1^, symmetrical stretching of C=C at 1577 cm^−1^, and C-H bending at 1060 cm^−1^, respectively^43, 44^.

**Figure 3:**
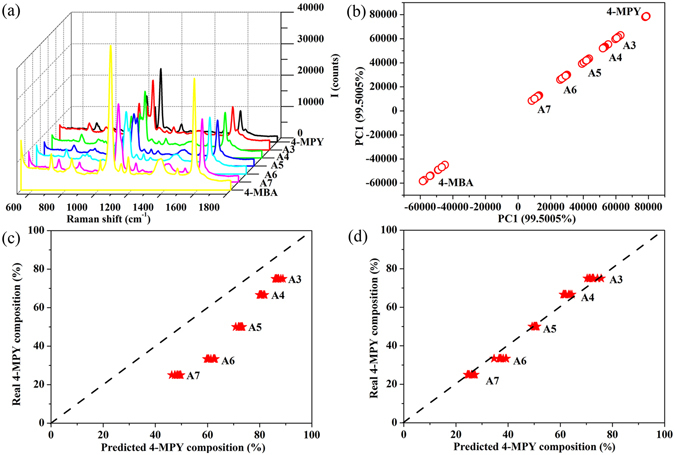
(**a**) SERS spectra of 4-MBA, 4-MPY, and their binary mixture sample A3 to A7. (**b**) Plot of PCA scores for 4-MBA, 4-MPY, and their binary mixture sample A3 to A7. (**c**) Plot of real composition in solution versus composition predicted by PCA. (**d**) Plot of real composition in solution versus composition of corrected PCA predictions.

Figure 3a shows SERS spectra of 1 × 10^–6^ M solution of 4-MPY, 4-MBA, and their binary mixture sample A3 to A7. Table 1 summarizes real composition in solution, composition predicted by PCA, adsorption kinetic factor for each component, and corrected PCA predictions for binary samples. As shown by Fig. 3a, features of mixtures are not obvious and the spectra of them are roughly similar. Though the tendency that SERS peaks of 1369, 1073 cm^−1^ assigned to 4-MBA decreased and peaks of 1094 cm^−1^ assigned to 4-MPY increased with incremental proportion of 4-MPY in binary samples can be observed, it is hard to do quantitative analysis directly from them. So, PCA analysis of the SERS spectra of 4-MPY and 4-MBA molecules were carried out and we got the loading matrix during the PCA modeling process. PCA is a method used for simplifying complex data sets based on building linear multivariate models using orthogonal basis vectors called principal components (PCs)^45^. In addition, PCA for the SERS spectra of all binary mixtures using the loading matrix were performed, and we plotted the scores of PC1 as presented in Fig. 3b. Figure 3b shows that results could be clustered into 7 separate groups and each group gathered together. It is obvious that each sample can be clearly recognized by PC1 which accounts for more than 99% of the variance. The SERS intensity of probe molecule is proportional positively related to their numbers adsorbed on the substrates, and PC1 are linearly related to the SERS intensity of this molecules. Hence, the mole fraction of each component adsorbed on the surface of substrates can be weighted by their PC1 scores. We averaged PC1 scores of 4-MPY, 4-MBA, and their binary samples, respectively. Table 1 shows mole fraction of each component calculated by PCA using the average scores. It is observed, that there is composition difference between the values calculated by PCA and the real ones, with error larger than 11%, even 27.56% for sample A6 which can’t be ignored. This comes from the difference in adsorption kinetics of each component, as to 4-MBA and 4-MPY mixtures solutions, 4-MBA adsorption capacities are not equal to that of 4-MPY. Hence, the composition of molecules adsorbed on Ag@Al_2_O_3_ nanorods as SERS substrate can be different from that in solution. Due to difference of adsorption kinetics of each component, we use adsorption kinetics factor *k* which represents the molecule’s adsorption capacity. Numerically, it is a ratio equals to composition predicted by PCA scores divided by that in solutions for each component. Results in Table 1 shows that the adsorption kinetics factor samples ranges from 1.16 to 1.91 for 4-MPY, and from 0.53 to 0.70 for 4-MBA, in all mixtures. This indicates that 4-MBA molecules have stronger adsorption ability as SERS substrates than that of 1, 4-BDT molecules in this condition. On the other hand, the adsorption kinetics factor for each component shows negligible variation that we consider them constant in all these mixtures. Although molecules’ adsorption ability is related to its concentration and solution environment, namely pH, competed molecules coexisted, etc., we assumed here that molecules present in mixtures at trace level are individuals and they don’t interact with other molecules. Hence, we use sample A5 as a reference, with which one could correct the prediction given by PCA. We can obtain other samples’ composition in solution by PCA combining with adsorption kinetics factor of sample A5, e.g., 1.44 for adsorption kinetics factor of 4-MPY, and 0.56 for adsorption kinetics factor of 4-MBA. Table 1 shows the normalized results considering the difference in molecules’ adsorption kinetics. It is found that for the five binary mixtures (i.e., sample A3 to A7) compositions obtained by correcting PCA predictions with sample A5 as the reference are very close to their real compositions in solution, with errors nearly less than 5%. To further elucidate this approach, we calculate the composition of 4-MPY by previously mentioned method with every spot plotted in Fig. 3b, and the results were shown in Fig. 3c. It is observed that results obtained by PCA prediction are far away from the dotted line y = x, which means there is composition discrepancy for each component between predicted value and real value in solution. For comparison, results that difference in adsorption kinetics of each component been considered in the prediction presented in Fig. 3d. Figure 3d shows that results obtained by correcting PCA prediction with adsorption kinetics factor of sample A5 are located around to the dotted line, which means compositions predicted are closer to real values in solution after correcting PCA predictions. Note that the dotted lines are only for guide of the eyes.

**Table 1:** Real composition in solution, composition predicted by PCA scores and errors, adsorption kinetics factor for each component, corrected PCA predictions, and errors for binary sample A3 to A7.

	A3	A4	A5	A6	A7
*real composition*					
X_4-MPY_:X_4-MBA_	3:1	2:1	1:1	1:2	1:3
4-MPY composition (%)	75	66.66	50	33.33	25
4-MBA composition (%)	25	33.33	50	66.66	75
*composition Predicted by PCA scores and errors*					
4-MPY Composition (%)	86.87	80.77	71.97	60.89	47.75
Error of 4-MPY Composition (%)	11.87	14.11	21.97	27.56	22.75
4-MBA Composition (%)	13.13	19.23	28.03	39.11	52.25
Error of 4-MBA Composition (%)	11.87	14.11	21.97	27.56	22.75
*adsorption kinetics factor for each component*					
k_4-MPY_	1.16	1.21	1.44	1.83	1.91
k_4-MBA_	0.53	0.58	0.56	0.59	0.70
*Corrected PCA predictions and errors*					
4-MPY Composition (%)	72.04	62.06	—	37.75	26.25
Error of 4-MPY Composition (%)	2.96	4.61	—	4.42	1.25
4-MBA Composition (%)	27.96	37.94	—	62.25	73.75
Error of 4-MBA Composition (%)	2.96	4.61	—	4.42	1.25

**Figure 4:**
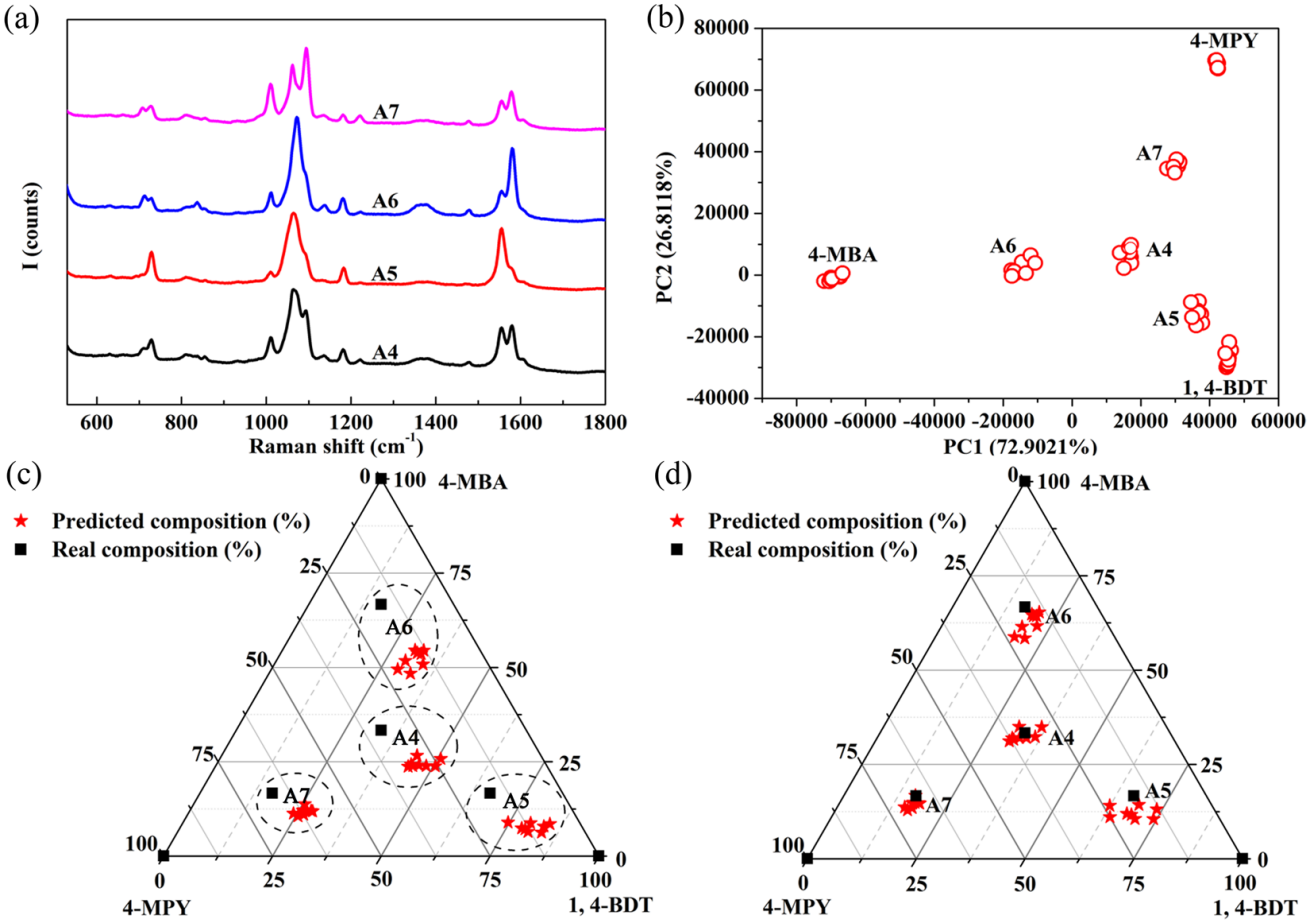
(**a**) SERS spectra of ternary mixture sample A4 to A7. (**b**) Plot of PCA scores for 1, 4-BDT, 4-MBA, 4-MPY, and their ternary mixture sample A4 to A7. (**c**) Plot of real composition in solution versus composition predicted by PCA. (**d**) Plot of real composition in solution versus composition of corrected PCA predictions.

To further verify this method, we made composition predictions for ternary mixtures and compared results with real values in solution. First, PCA was carried out for 1, 4-BDT, 4-MBA, and 4-MPY molecules using their SERS spectra to get the loading matrix. Table 2 summarizes real composition in solution, composition predicted by PCA, adsorption kinetic factor for each component, and corrected PCA predictions for ternary sample A4 to A7. Figure 4a shows samples’ typical SERS spectra. PCA for these ternary mixtures spectra were performed with loading matrix and we obtained PCs scores for the ternary mixtures which were plotted in Fig. 4b. It is observed that two PCs (PC1 and PC2) accounts for ~99.71% of the variance. It is obvious that the PCA scores also gathered together. We averaged PC1 and PC2 scores of 1, 4-BDT, 4-MPY, 4-MBA, and their ternary mixtures, respectively. And we predicted the composition of each sample by using average PC1 and PC2 scores as shown in Table 2. However, there still exist errors between predicted compositions and real values due to the difference in adsorption kinetics of each component, e.g., 13.89% for 1,4-BDT in A4 samples, 12.75% for 1,4-BDT in A5 samples, 15.14% for 4-MBA in A6 samples. Hence, we calculated adsorption kinetics factor for each component in the similar way as we did for binary mixtures (presented in Table 2). Result indicates that the adsorption kinetics factors for a specific component show minor changes. Hence, we obtained other samples’ compositions by correcting PCA predictions using adsorption kinetics factors of sample A4, e.g., 1.42 for adsorption kinetics factor of 1,4-BDT, 0.75 for adsorption kinetics factor of 4-MBA, and 0.83 for adsorption kinetics factor of 4-MPY. It is noticeable that the predicted compositions shown in Table 2 are in good agreement with the real ones, i.e., the errors are around and less than 5%. In addition, we obtained composition of samples (spots plotted in Fig. 4b) by PCA prediction. Plot of real composition in solution versus composition predicted by PCA was presented in Fig. 4c. For comparison, corrected PCA predictions according to adsorption kinetics factor of sample A4 were plotted in Fig. 4d. Figure 4d illustrates that the results obtained by correcting PCA predictions using adsorption kinetic factors of sample A4 are located around real values in solution, which proves the accuracy of prediction. On the other hand, results neglecting adsorption kinetics difference of each molecule are far away from the real values in solution.

**Table 2:** Real composition in solution, composition predicted by PCA scores and errors, adsorption kinetics factor for each component, corrected PCA predictions, and errors for ternary sample A4 to A7.

	A4	A5	A6	A7
*real composition*				
X_1, 4-BDT_:X_4-MBA_:X_4-MPY_	1:1:1	4:1:1	1:4:1	1:1:4
1, 4-BDT composition (%)	33.33	66.67	16.67	16.67
4-MBA composition (%)	33.33	16.67	66.67	16.67
4-MPY composition (%)	33.33	16.67	16.67	66.67
*composition Predicted by PCA scores and errors*				
1, 4-BDT Composition (%)	47.22	79.42	30.83	26.10
Error of 1, 4-BDT Composition (%)	13.89	12.75	14.16	9.43
4-MBA Composition (%)	25.00	7.64	51.53	11.74
Error of 4-MBA Composition (%)	8.33	9.03	15.14	4.93
4-MPY Composition (%)	27.78	12.94	17.64	62.16
Error of 4-MPY Composition (%)	5.55	3.73	0.97	4.51
*adsorption kinetics factor*				
k_1,4-BDT_	1.42	1.19	1.85	1.56
k_4-MBA_	0.75	0.46	0.77	0.70
k_4-MPY_	0.83	0.78	1.06	0.93
*Composition by correcting PCA predictions and errors*				
1, 4-BDT Composition (%)	—	68.56	19.49	16.95
Error of 1, 4-BDT Composition (%)	—	1.89	2.82	0.28
4-MBA Composition (%)	—	12.46	61.54	14.40
Error of 4-MBA Composition (%)	—	4.21	5.12	2.26
4-MPY Composition (%)	—	18.99	18.96	68.64
Error of 4-MPY Composition (%)	—	2.32	2.29	1.97

**Figure 5:**
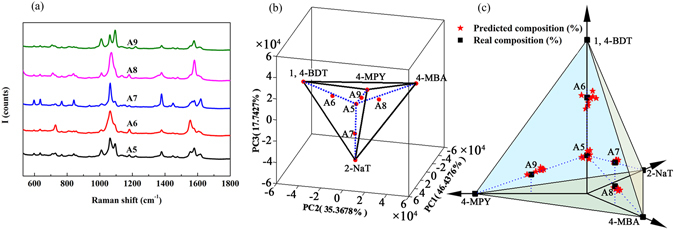
(**a**) SERS spectra of quaternary mixture sample A5 to A9. (**b**) Plot of PCs scores for 1, 4-BDT, 2-NaT, 4-MBA, 4-MPY, and their quaternary mixtures. (**c**) Plot of real composition in solution versus composition of corrected PCA predictions.

This method of predicting real compositions in solution was also carried out for 1, 4-BDT, 2-NaT, 4-MBA, 4-MPY, and their quadruple mixtures. Table S1 (provided in Supporting Information) summarizes real composition in solution, composition predicted by PCA, adsorption kinetic factor for each component, and corrected PCA prediction of quadruple samples. Figure 5a shows their quadruple mixtures’ SERS spectra. One can observe that SERS spectra were roughly similar. Hence, we perform PCA for 1, 4-BDT, 2-NaT, 4-MPY, and 4-MBA molecules to get loading matrix. Subsequently, PCA for the SERS spectra of quadruple mixture samples using the loading matrix were performed, and the average PCs scores of each samples were presented in Fig. 5b. It shows that three PCs (PC1, PC2 and PC3) accounts for around 99.55% of the variance. Compositions of mixture samples (presented in Table S1) were calculated by PCA using the average PCs scores mentioned above. Similarly, composition errors can be observed between values calculated by PCA and the real ones in solution due to molecules’ different adsorption kinetics, e.g., 12.91% for 4-MPY in sample A5 and 17.95% for 4-MBA in sample A8. We obtained adsorption kinetic factor of each component in all quaternary samples the way mentioned previously and it shows minor changes. Then, the adsorption kinetics factor for each component of sample A5 was treated as the reference and corrected predictions were presented in Table S1. The errors are around and less than 6%. Furthermore, we corrected PCA predictions of every quadruple mixtures spectrum collected by using the adsorption kinetics factor of sample A5 and results were presented in Fig. 5c. It shows that the predicted spots are located around real ones, which demonstrated that this method is also applicable for quadruple mixtures.

To further confirm this method, we made the same process for the remaining five binary mixtures and three ternary mixtures which is two or three of 1, 4-BDT, 2-NaT, 4-MPY, and 4-MBA. The real compositions of binary and ternary mixtures are the same shown by Table 1 or Table 2. Corrected predictions were based on the adsorption kinetics factors of the reference sample and results are presented in Supporting Information (Figure S1 and S2). As shown by Figure S1 and S2, the predicted composition of each component is located around their real composition in solution which indicates good validity of this method.

Subsequently, in order to confirm the stability of the substrates, we performed XPS again after we obtained SERS spectrum of sample A5 in quadruple mixtures. Results are shown in Supporting Information (Figure S3). It can be observed that typical metallic properties are maintained as the peaks of doublet Ag3d_5/2_ and Ag3d_3/2_ located at 368.1 eV and 374.1 eV, respectively. It also suggested the excellence stability of our substrates.

To extend this method to wider range of applications, a general calculation formula of predicting mixtures composed of n + 1 component were proposed as given below:

$$[\begin{array}{ccc}({x}_{1,1}-{x}_{m,1}){k}_{1}^{\ast } & \cdots  & ({x}_{n+1,1}-{x}_{m,1}){k}_{n+1}^{\ast }\\ \vdots  & \ddots  & \vdots \\ \begin{array}{c}({x}_{1,n}-{x}_{m,n}){k}_{1}^{\ast }\\ 1\end{array} & \begin{array}{c}\cdots \\ \ldots \end{array} & \begin{array}{c}({x}_{n+1,n}-{x}_{m,n}){k}_{n+1}^{\ast }\\ 1\end{array}\end{array}]\,[\begin{array}{c}{a}_{1}\\ \vdots \\ \begin{array}{c}{a}_{n}\\ {a}_{n+1}\end{array}\end{array}]=[\begin{array}{c}0\\ \vdots \\ \begin{array}{c}0\\ 1\end{array}\end{array}]$$

where the PC_n_ score of the m component is *x*_*(m,n)*_, *a*_*n*_ is composition and *k*_n_^*^ is adsorption kinetics factor of the n component in solution, respectively. In multi-component system, *x*_*(m,n)*_ value can be given by PCA. Therefore, we can obtain compositions in solution based on adsorption kinetics factor for each component of reference sample.

## Conclusions

In conclusion, the current study provides a simple reliable way to predict chemical compositions for trace-level multi-component mixture solutions. Using the reference’s adsorption kinetics factor for each component, we can correct the predictions given by PCA, within acceptable errors. We demonstrated the validity by predicting binary, ternary and quaternary mixtures of 1, 4-BDT, 2-NAT, 4-MPY, and 4-MBA in solution. We also proposed a calculation formula which is applicable complex mixtures. This new technique provides a good opportunity for quantitative analysis of chemical mixtures using SERS at trace levels.

## Methods

### Chemicals

1, 4-Benzenedithiol (1, 4-BDT), 2-Naphthalenethiol (2-NaT), 4-Mercaptobenzoic acid (4-MBA), and 4-Mercaptopyridine (4-MPY) were purchased from J&K Scientific Ltd., Beijing, China. Ethanol was purchased from Beijing Chemical Glass station Co. Ltd., China. All chemicals were used without purification.

### Synthesis of Ag NRs

Slanted Ag nanorods were fabricated by oblique angle deposition using an electron-beam system (GLAD, Thermionics Inc.) that has been described previously^46, 47^. Ag NRs were prepared on Si (001) substrates with a background vacuum level of 10^−6^ Pa. During deposition, the incident angle of Ag beam was 86° with respect to surface normal of substrates at a deposition rate of 0.75 nm/s. The deposition process completes when quartz crystal microbalance (QCM) shows reading of 1000 nm.

### Synthesis of Ag@Al_2_O_3_

Al_2_O_3_ films were coated on as-prepared Ag NRs in the ALD system (MNT-100, Wuxi MNT Micro and Nanotech Co.) as reported previously^48, 49^. The film growth was conducted at 70 °C using trimethyl-aluminum (TMA, maintained at 150 °C) as the precursor for alumina (Al) and water (maintained at 40 °C) as the precursor for oxygen. The ALD sequence consisted of pulsing of trimethylaluminum followed by nitrogen purging followed by pulsing of water and nitrogen purging. During one ALD cycle, the pulsing time for trimethylaluminum were maintained at 20 ms and the nitrogen purging time was at 10 s, while water were maintained at 10 ms and the nitrogen purging time was 20 s. The reaction was conducted for 1 cycle over Ag NRs.

### Preparation of analytes and SERS detections

1, 4-BDT, 2-NaT, 4-MPY, and 4-MBA were dissolved in ethanol and diluted to 1 × 10^−6^ M, respectively. For chemical mixtures, the total concentrations of chemical mixtures were all 1 × 10^−6^ M. The SERS measurements were conducted with an optical fiber micro-Raman system (i-Raman Plus, B&W TEK Inc.), using a 785 nm laser as the excitation source. Before Raman spectra of probes were acquired, all substrates were immersed into different solutions for 20 minutes to make the probes adsorbed, washed thoroughly by ethanol to remove the residual molecules, and dried naturally in air. The SERS spectrum was collected from 8 points randomly selected on each sample using 90 mW laser power with a collection time of 10 s.

## Electronic supplementary material

Supporting information
