# Supplementary material for: Semi-quantitative analysis of multiple chemical mixtures in solution at trace level by surface-enhanced Raman Scattering
Source: Sci Rep. 2017 Jul 21;7:6186. doi: 10.1038/s41598-017-06543-y (PMC5522419; doi:10.1038/s41598-017-06543-y)
Supplement: Supplementary file 1 — Supporting information [file 41598_2017_6543_MOESM1_ESM.pdf]

Supporting information:

Semi-quantitative analysis of multiple chemical mixtures in solution at trace level by surface-enhanced Raman Scattering

Sumeng Zou, Mengjing Hou, Jianghao Li, Lingwei Ma, and Zhengjun Zhang

* **Corresponding author**: Zhengjun Zhang, Tel.: +8601062797033, E-mail: zjzhang@tsinghua.edu.cn, Postal address: Yifu technology and science building, Tsinghua University, Hai Dian district, Beijing.

Table for quaternary mixtures

Table S1. Real composition in solution, composition predicted by PCA and errors, adsorption kinetics factor for each component, and corrected PCA predictions and errors of quadruple sample A5 to A9.

	A5	A6	A7	A8	A9
real composition					
$X_{1,4\text{-BDT}}$:$X_{2\text{-NaT}}$:$X_{4\text{-MBA}}$:$X_{4\text{-MPY}}$	1:1:1:1	5:1:1:1	1:5:1:1	1:1:5:1	1:1:1:5
1, 4-BDT composition (%)	25	62.5	12.5	12.5	12.5
2-NaT composition (%)	25	12.5	62.5	12.5	12.5
4-MBA composition (%)	25	12.5	12.5	62.5	12.5
4-MPY composition (%)	25	12.5	12.5	12.5	62.5
composition Predicted by PCA scores and errors					
1, 4-BDT composition (%)	23.49	56.91	12.92	11.91	11.22
Error of 1, 4-BDT Composition (%)	1.51	5.59	0.42	0.59	1.18
2-NaT composition (%)	24.23	16.19	64.65	19.53	13.18
Error of 4-NaT Composition (%)	0.77	3.69	2.15	7.03	0.68
4-MBA composition (%)	14.36	7.08	6.80	44.55	5.83
Error of 4-MBA Composition (%)	10.64	5.42	5.7	17.95	6.67
4-MPY composition (%)	37.91	19.82	15.63	24.02	69.77
Error of 4-MPY Composition (%)	12.91	7.32	3.13	11.52	7.27
adsorption kinetics factor					
$K_{1,4\text{-BDT}}$	0.94	0.91	1.03	0.95	0.90
$k_{2\text{-NaT}}$	0.97	1.30	1.03	1.56	1.05
$k_{4\text{-MPY}}$	0.57	0.57	0.54	0.71	0.47
$k_{4\text{-MBA}}$	1.52	1.58	1.25	1.92	1.12
Composition by correcting PCA predictions and errors					
1, 4-BDT composition (%)	-	58.99	13.40	10.04	14.62
Error of 1, 4-BDT Composition (%)	-	3.51	0.90	2.46	2.12
2-NaT composition (%)	-	16.27	65.01	15.96	16.64
Error of 4-NaT Composition (%)	-	3.77	2.51	3.46	4.14
4-MBA composition (%)	-	12.00	11.54	61.44	12.42
Error of 4-MPA Composition (%)	-	0.49	0.96	1.06	0.08
4-MPY composition (%)	-	12.73	10.05	12.55	56.32
Error of 4-MBY Composition (%)	-	0.23	2.45	0.05	6.18

Figures for predicting binary mixtures in solution

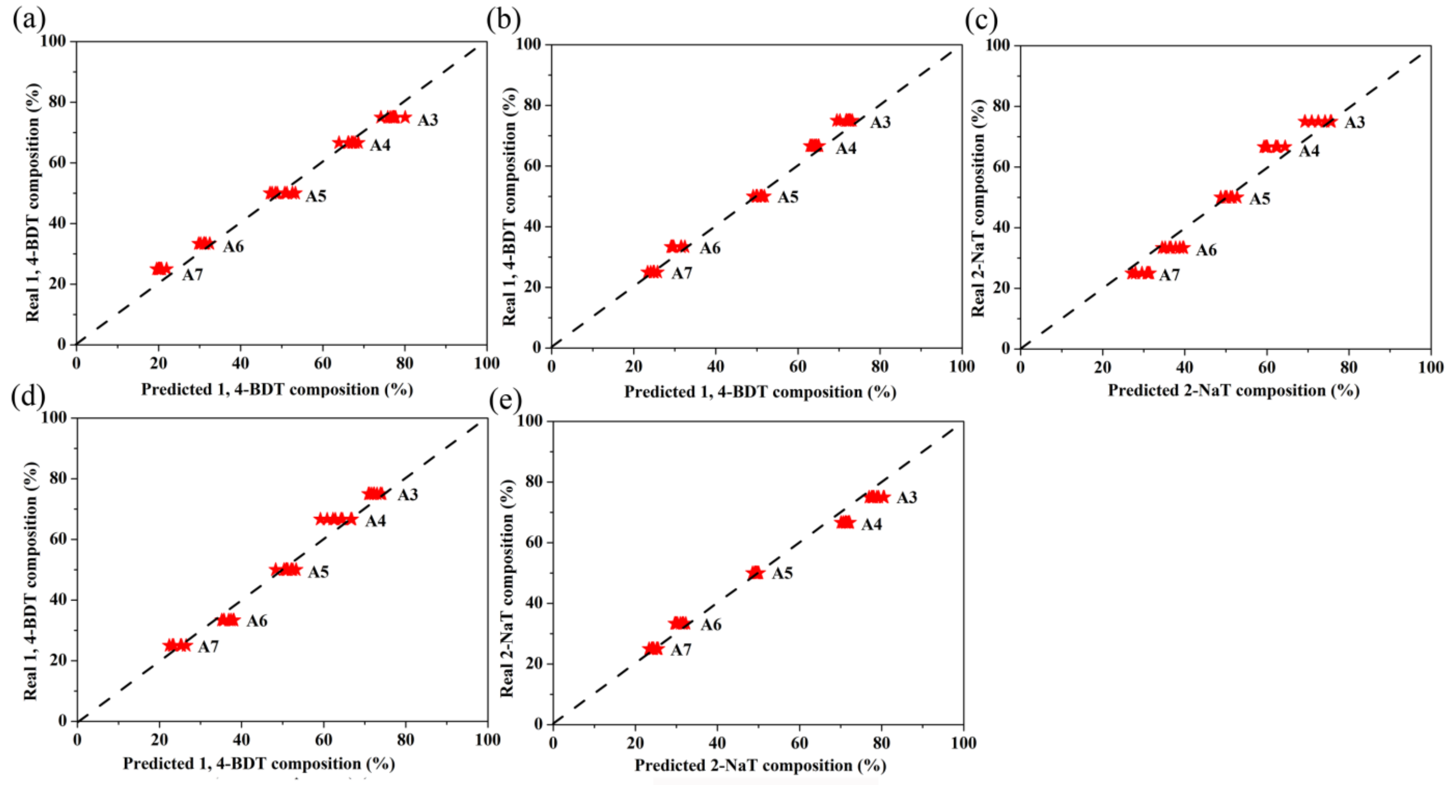

Figure S1. Plots of real composition in solution versus composition of corrected PCA predictions for (a) binary mixture of 1, 4-BDT and 4-MBA, (b) binary mixture of 1, 4-BDT and 2-NaT, (c) binary mixture of 2-NaT and 4-MBA, (d) binary mixture of 1, 4-BDT and 4-MPY, and (e) binary mixture of 2-NaT and 4-MPY.

Figures for predicting ternary mixtures in solution

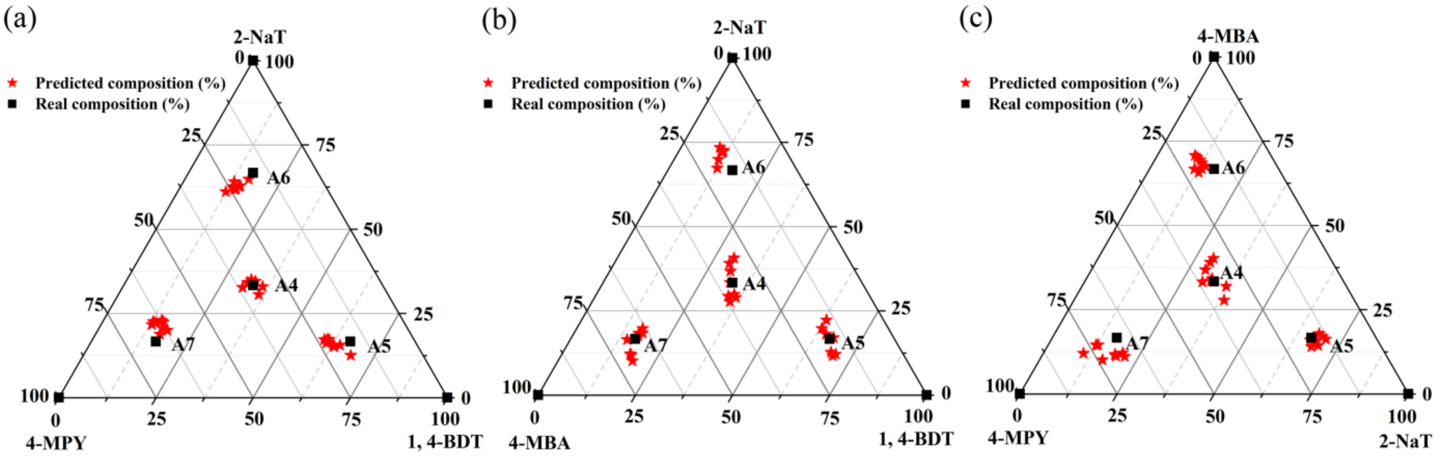

Figure S2. Plots of real composition in solution versus composition of corrected PCA predictions for (a) ternary mixture of 1, 4-BDT, 2-NaT, and 4-MPY, (b) ternary mixture of 1, 4-BDT, 2-NaT, and 4-MBA, and (c) ternary mixture of 2-NaT, 4-MBA, and 4-MPY.

Figure for XPS of $Ag@Al_2O_3$ nanorods after study

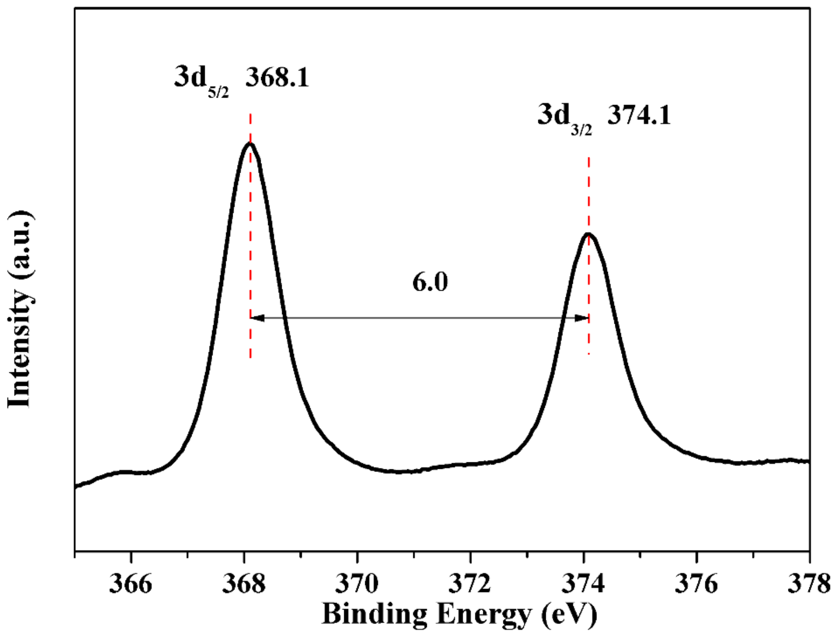

Figure S3. Ag $3d_{5/2}$ and Ag $3d_{3/2}$ XPS spectra of $Ag@Al_2O_3$ nanorods after SERS acquisition for sample A5 in quadruple mixtures.
